# Supplementary material for: LRRK2 p.G2385R and p.R1628P variants in a multi-ethnic Asian Parkinson’s Cohort: epidemiology and clinical insights
Source: NPJ Parkinsons Dis. 2025 Nov 18;11:320. doi: 10.1038/s41531-025-01166-x (PMC12627830; doi:10.1038/s41531-025-01166-x)
Supplement: Supplementary file 1 — Supplementary Information [file 41531_2025_1166_MOESM1_ESM.pdf]

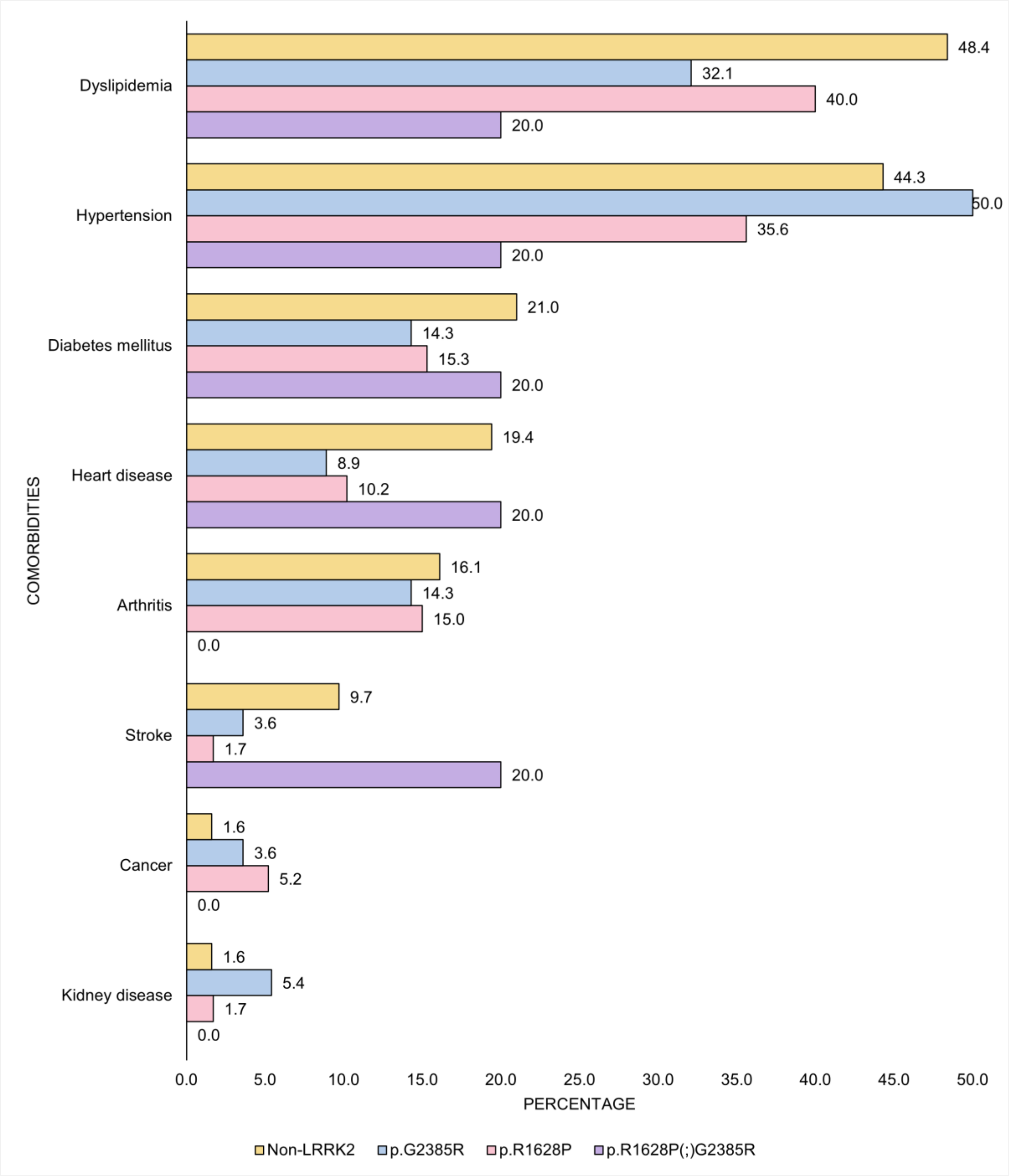

Supplementary Figure 1. Frequency of comorbidities in PD patients with and without *LRRK2* Asian risk variants (p.G2385R and p.R1628P) (n=185). There was only one *LRRK2* p.R1628P PD patient diagnosed with inflammatory bowel disease.

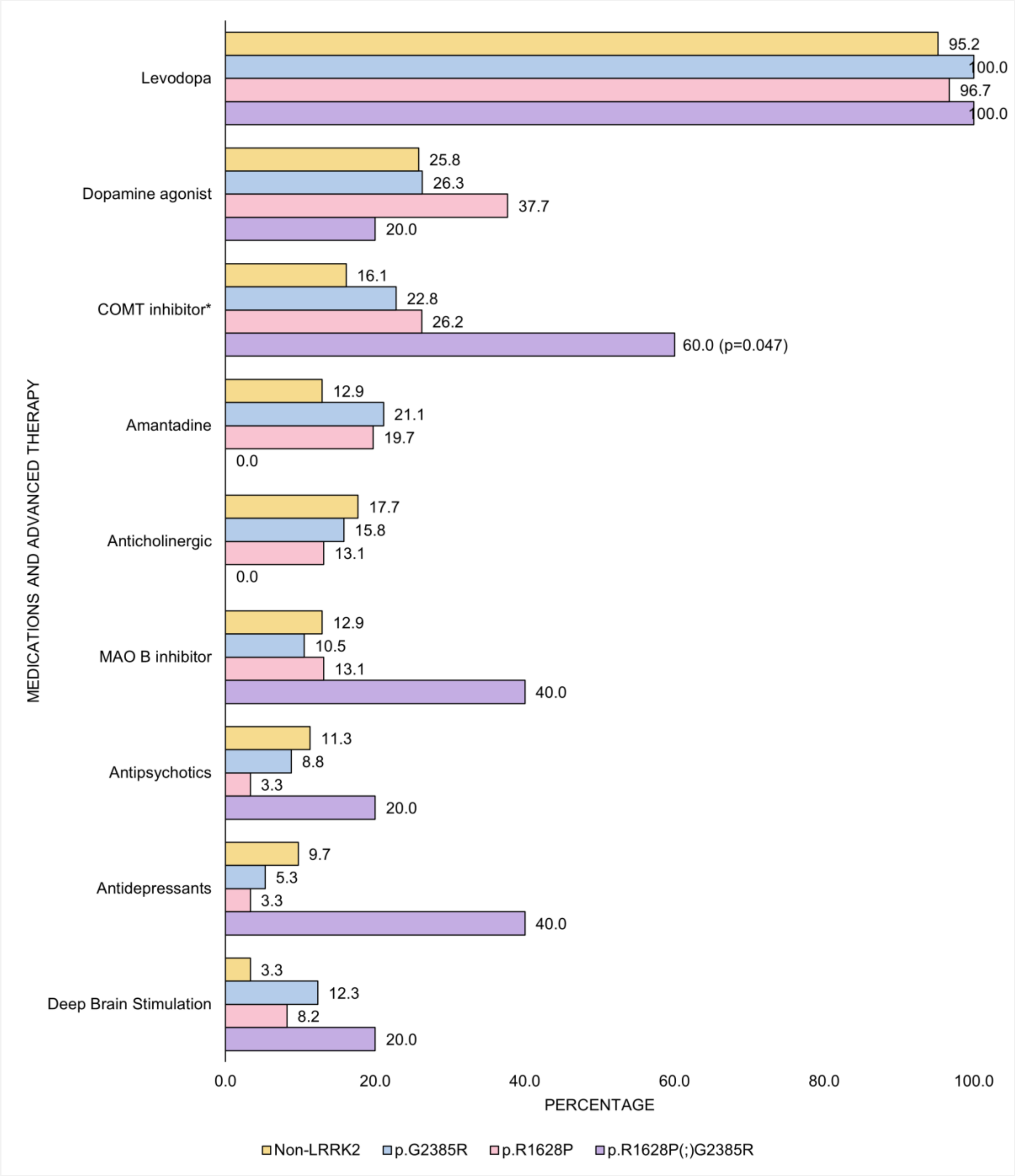

Supplementary Figure 2. Treatment profile (oral medications and advanced therapies) in PD patients with and without *LRRK2* Asian risk variants (p.G2385R and p.R1628P) (n=185). There was only one patient (non-*LRRK2* PD) on apomorphine infusion. Significant differences between groups were denoted by an asterisk (*). Abbreviations used in the figure include: COMT (Catechol-O-methyltransferase), MAO-B (Monoamine Oxidase B).
